# Let’s doff: a gown conservation strategy for multidrug-resistant organism colonization during the COVID-19 pandemic and beyond

**DOI:** 10.1017/ash.2025.10069

**Published:** 2025-07-28

**Authors:** Kelsey L Rowe, Josephine Fox, Carole Leone, Lydia J Grimes, Kenneth Whalen, David K Warren, Jonas Marschall

**Affiliations:** 1 Washington University School of Medicine, St. Louis, MO, USA; 2 Saint Louis Children’s Hospital, St. Louis, MO, USA; 3 Barnes-Jewish Hospital, St. Louis, MO, USA; 4 BJC Healthcare, St. Louis, MO, USA; 5 University of Arizona College of Medicine, Phoenix, AZ, USA

## Abstract

A COVID-19 pandemic gown conservation strategy for methicillin-resistant *Staphylococcus aureus* (MRSA) and vancomycin-resistant enterococci (VRE) asymptomatically colonized patients caused no significant difference in healthcare-associated MRSA (HA-MRSA) bacteremia, healthcare-associated VRE (HA-VRE) bacteremia, or healthcare-associated *Clostridioides difficile* infections (HA-CDI) versus prepandemic contact precautions (CP). Postpandemic HA-VRE and HA-CDI rates mirrored national trends.

## Introduction

The COVID-19 pandemic caused extreme personal protective equipment (PPE) shortages. A survey of over 20,000 health care workers (HCWs) in March through May of 2020 showed 22% of HCWs reported “sometimes” or “always” lacking appropriate PPE.^1^ Due to these shortages, hospitals sought methods to safely conserve PPE.

The Healthcare Infection Control Practices Advisory Committee (HICPAC) has traditionally recommended contact precautions (CP) with gowns and gloves in acute care settings for patients colonized or infected with multidrug-resistant organisms (MDROs).^2^ However, a 2018 meta-analysis found that discontinuation of CP for methicillin-resistant *Staphylococcus aureus (*MRSA) and vancomycin-resistant enterococci (VRE) was not associated with increased infection rates. Counterintuitively, removal of CP resulted in MRSA infections trending downward, and VRE infections were significantly reduced.^3^ Therefore, modifying CP for MDRO colonized patients came to be considered a viable option for PPE conservation during the COVID-19 pandemic.

This study aimed to evaluate a PPE conservation strategy that modified CP for patients asymptomatically colonized with MRSA and VRE and determine its impact on healthcare-associated MRSA (HA-MRSA) bacteremia, healthcare-associated *Clostridioides difficile* infections (HA-CDI), and healthcare-associated VRE (HA-VRE) infections.

## Methods

This study was performed at BJC HealthCare, a 14-site hospital group with more than 3,000 acute care beds located in Missouri and Illinois. Colonized patients were identified by prior positive MRSA or VRE cultures without current infections at that site. Some BJC hospitals also identified colonized patients via admission surveillance cultures (MRSA nasal swabs and VRE stool or rectal swabs) for high-risk populations. Surveillance cultures were paused during modified CP. Patients with active MRSA, *Clostridioides difficile*, or VRE infections were excluded from modified CP.

Prior to 2020 at BJC HealthCare, baseline CP for MRSA or VRE colonization involved single-use gowns and gloves, consistent with HICPAC guidelines.^2^ We defined “modified CP” as HCWs donning gloves upon entry into rooms of patients colonized with MRSA or VRE, but foregoing gowns unless needed for standard precautions.

Cases of HA-MRSA bacteremia and HA-CDI were based on National Healthcare Safety Network (NHSN) laboratory identification (LabID) criteria, defined as a positive laboratory result more than three days after admission. Cases of HA-VRE bacteremia and bacteriuria were defined as a positive culture more than two days after admission. Rates were retrospectively identified using available Electronic Health Record data across acute care beds in the BJC HealthCare system.

BJC HealthCare initiated modified CP in April 2020 and announced the return to baseline CP in July 2021. Therefore, the study was divided into three periods: prepandemic baseline CP in 2019, pandemic modified CP in 2021, and postpandemic baseline CP in 2023. To minimize temporal bias, January through August was analyzed in all three periods. Rates of infection per 1,000 patient-days were compared using MedCalc two-way *χ* ^2^ analyses.^4^

## Results

HA-MRSA bacteremia rates were not significantly different across all periods. The HA-MRSA bacteremia rate with baseline CP in 2019 was 0.070 (36 cases/516,390 patient-days), compared to a rate of 0.062 (34/545,086) with modified CP in 2021 and a rate of 0.069 (40/577,095) with baseline CP in 2023 (*P* = .87).

The rate of HA-CDI with modified CP in 2021 was 0.362 (182 cases/503,327 patient-days), which was not significantly different from the rate with baseline CP in 2019, 0.393 (186/473,884) (*P* = .43), or from the rate with baseline CP in 2023, 0.297(159/535,901) (*P* = .07). However, the rate of HA-CDI with baseline CP in 2023 was significantly lower than the rate with baseline CP in 2019 (*P* = .009).

HA-VRE bacteremia rates were not significantly different across all periods. The rate of HA-VRE bacteremia was 0.038 (19 cases/493,794 patient-days) with baseline CP in 2019 compared to a rate of 0.047 (24/510,814) with modified CP in 2021 and a rate of 0.057 (31/547,614) with baseline CP in 2023 (*P* = .41).

The rate of HA-VRE bacteriuria with baseline CP in 2019 was 0.059 (29 cases/493,794 patient-days), which was not significantly different from the rate with modified CP in 2021, 0.069 (35/510,814) (*P* = .54). However, the rate of HA-VRE bacteriuria with baseline CP in 2023 was 0.108 (59/547,614), which was significantly higher than the rate with modified CP in 2021 (*P* = .03) and significantly higher than the rate with baseline CP in 2019 (*P* = .007).

**Figure 1.**
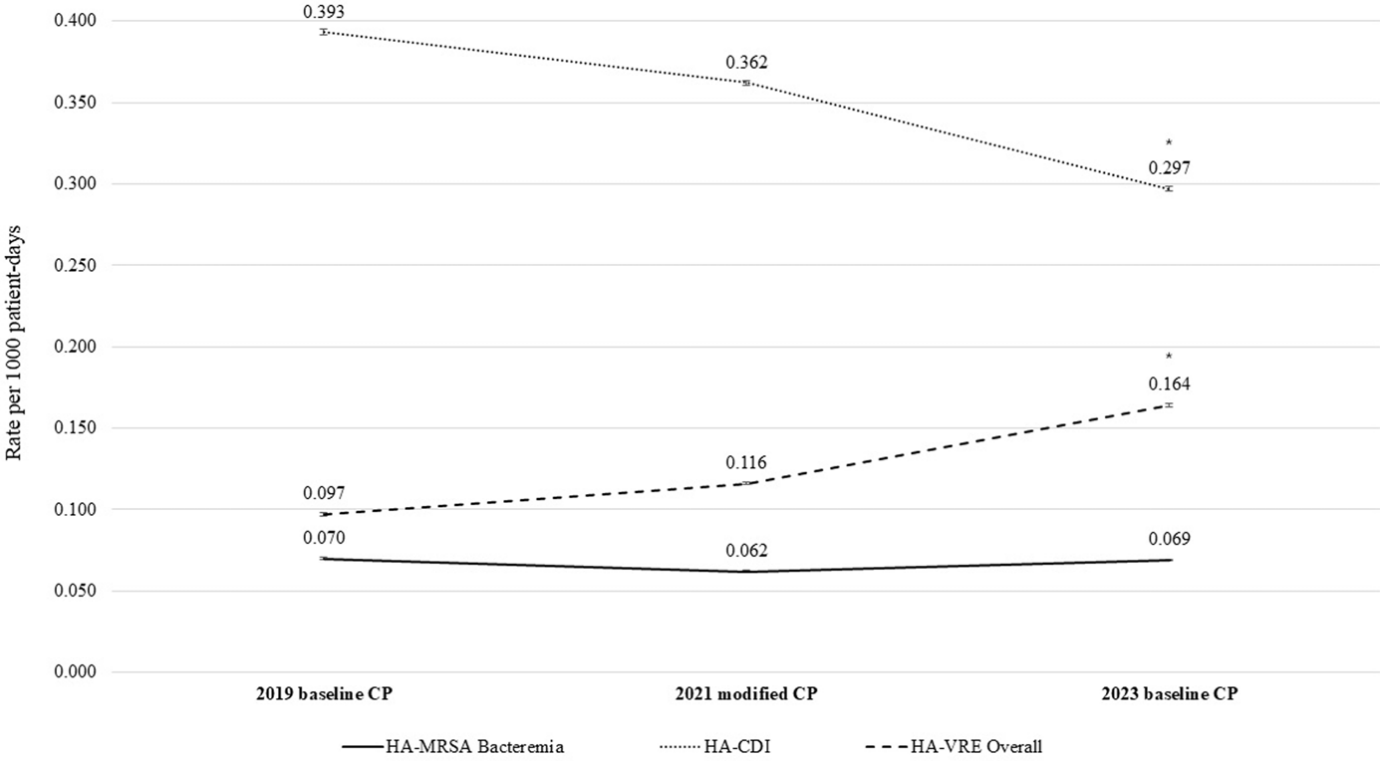
National Healthcare Safety Network (NHSN) laboratory identification (LabID) healthcare-associated methicillin-resistant *Staphylococcus aureus* (HA-MRSA) bacteremia, LabID healthcare-associated *Clostridioides difficile* infection (HA-CDI), and healthcare-associated vancomycin-resistant enterococci (HA-VRE) infection rates per 1,000 patient-days with baseline contact precautions (CP) in 2019, modified CP in 2021, and baseline CP in 2023.

The overall rate of HA-VRE infections was 0.097 (48 cases/493,794 patient-days) with baseline CP in 2019, which was not significantly different from the rate with modified CP in 2021, 0.116 (59/510,814) (*P* = .37). However, the rate of HA-VRE infections with baseline CP in 2023 was 0.164 (90/547,614), which was significantly higher than the rate with modified CP in 2021 (*P* = .03) and significantly higher than the rate with baseline CP in 2019 (*P* = .003) (Table 1, Figure 1).

**Table 1.** Rates of National Healthcare Safety Network (NHSN) laboratory identification (LabID) healthcare-associated methicillin-resistant *Staphylococcus aureus* (HA-MRSA) bacteremia, LabID healthcare-associated *Clostridioides difficile* (HA-CDI), and healthcare-associated vancomycin-resistant enterococci (HA-VRE) infections during baseline contact precautions (CP) in 2019, modified CP in 2021, and baseline CP in 2023, per 1,000 patient-days with 95% Confidence Intervals (CI) and χ2 *P* values

Healthcare-Associated Infection	2019		2021		2023		P values			
	Rate	95% CI	Rate	95% CI	Rate	95% CI	Overall	2019 vs 2021	2021 vs 2023	2019 vs 2023
HA-MRSA Bacteremia	.070 (36/516,390)	[.069–.070]	.062 (34/545,086)	[.062–.063]	.069 (40/577,095)	[.069–.007]	.87†			
HA-CDI	.393 (186/473,884)	[.391–.394]	.362 (182/503,327)	[.360–.363]	.297 (159/535,901)	[.295–.298]	.03*	.43	.07	.009*
HA-VRE Bacteremia	.038 (19/493,794)	[.038–.039]	.047 (24/510,814)	[.046–.048]	.057 (31/547,614)	[.056–.057]	.41†			
HA-VRE Bacteriuria	.059 (29/493,794)	[.058–.059]	.069 (35/510,814)	[.068–.069]	.108 (59/547,614)	[.107–.109]	.01*	.54	.03*	.007*
HA-VRE Overall	.097 (48/493,794)	[.096–.098]	.116 (59/510,814)	[.115–.116]	.164 (90/547,614)	[.163–.165]	.007*	.37	.03*	.003*

Statistically significant with *P* < .05.

†Overall *χ* ^2^ *P* values did not show significant difference between any year.

## Discussion

During the COVID-19 pandemic, our hospital group modified CP for patients colonized with MRSA or VRE as a gown conservation effort. Modified CP were not associated with increased rates of HA-MRSA bacteremia, HA-CDI, or HA-VRE infections compared to prepandemic rates with baseline CP.

Our results support existing literature that baseline CP for some MDRO colonized patients may be a low-value intervention. A 2019 literature review concluded that CP have not been shown to effectively reduce transmission of MRSA and VRE.^5^ Additionally, isolation may affect patient safety and experience. A 2010 systematic review showed isolated patients had higher scores for depression and anxiety, higher anger-hostility scores, and reports of fear and loneliness. Isolated patients also experienced more medical errors, more adverse events, and less frequent care documentation, however, these outcomes were not adjusted for severity of illness.^6^

Furthermore, PPE may have a significant global cost and environmental impact. The National Health System (NHS) in the United Kingdom reported that PPE was the third largest health expense during the COVID-19 pandemic, costing £15 billion between 2020 and 2022.^7^ PPE use by the NHS in the first 6 months of the pandemic also resulted in over 100,000 tons of CO2 emissions according to one life-cycle analysis, with gowns having the highest carbon footprint per item.^8^ Thus, discontinuation of CP for MDRO colonization could reduce PPE costs and emissions. Supplies and labor costs for active surveillance could also be eliminated.

This study showed significant reduction in HA-CDI in the postpandemic baseline CP period, however this mirrors national rates. The CDC reported a 13% decrease in HA-CDI in acute care hospitals between 2022 and 2023.^9^ Additionally, in 2022 BJC implemented measures to reduce repeat CDI testing. The BJC laboratory also stopped reflexing specimens with negative *Clostridioides difficile* toxin EIA and positive glutamate dehydrogenase test results to PCR. These efforts likely reduced HA-CDI rates at BJC in 2023. This study also showed a significant increase in HA-VRE bacteriuria and overall HA-VRE infection rates in the postpandemic period. This reflects national trends, too, as CDC data showed increased HA-VRE from 2019 to 2022, which could be related to changing antibiotic prescribing practices.^10^

Limitations of this study are that PPE compliance audits were not performed, and enhanced cleaning during the COVID-19 pandemic may have affected results. The three short study periods may have also been insufficient to detect infection rate differences.

This study is significant because it highlights how a PPE conservation strategy was employed successfully during the COVID-19 pandemic without increasing HA-MRSA bacteremia, HA-CDI, or HA-VRE infections. Due to this analysis, our hospital system is modifying MDRO isolation practices permanently to improve patient experience and reduce PPE cost and environmental impact.

## References

[1] Rich-Edwards JW , Ding M , Rocheleau CM , Boiano JM , Kang JH , Becene I, et al. American Frontline Healthcare Personnel’s Access to and Use of Personal Protective Equipment Early in the COVID-19 Pandemic. J Occup Environ Med 2021;63:913–2020. 34238908 10.1097/JOM.0000000000002308 PMC8562916

[2] Siegel JD , Rhinehart E , Jackson M , Chiarello L. Health Care Infection Control Practices Advisory C. Guideline for isolation precautions: preventing transmission of infectious agents in health care settings. Am J Infect Control 2007;35:S65–164. 10.1016/j.ajic.2007.10.007 PMC7119119 18068815

[3] Marra AR , Edmond MB , Schweizer ML , Ryan GW , Diekema DJ. Discontinuing contact precautions for multidrug-resistant organisms: a systematic literature review and meta-analysis. Am J Infect Control 2018;46:333–40. 29031432 10.1016/j.ajic.2017.08.031

[4] MedCalc Software Ltd. Two-way chi-squared test. https://www.medcalc.org/calc/chisquared-2way.php Version 23.0.8. Accessed November 21, 2024.

[5] Young K , Doernberg SB , Snedecor RF , Mallin E. Things we do for no reason: contact precautions for MRSA and VRE. J Hosp Med 2019;14:178–8080. 30811326 10.12788/jhm.3126

[6] Abad C , Fearday A , Safdar N. Adverse effects of isolation in hospitalised patients: a systematic review. J Hosp Infect 2010;76:97–102. 20619929 10.1016/j.jhin.2010.04.027 PMC7114657

[7] Stiebahl S. Funding and Expenditure. United Kingdom parliament house of commons library website. https://researchbriefings.files.parliament.uk/documents/SN00724/SN00724.pdf. Published 2024. Accessed November 21, 2024.

[8] Rizan C , Reed M , Bhutta MF. Environmental impact of personal protective equipment distributed for use by health and social care services in England in the first six months of the COVID-19 pandemic. J R Soc Med 2021;114:250–63. 33726611 10.1177/01410768211001583 PMC8150566

[9] National and State Healthcare-Associated Infections Progress Report. Centers for Disease Control and Prevention Antimicrobial Resistance and Patient Safety Portal website. https://arpsp.cdc.gov/profile/national-progress-2023/united-states. Published 2024. Accessed November 21, 2024.

[10] Antimicrobial Resistance Threats in the United States, 2021-2022. Centers for Disease Control and Prevention website. https://www.cdc.gov/antimicrobial-resistance/data-research/threats/update-2022.html. Published 2024. Accessed November 21, 2024.

